# Faecal immunochemical test is superior to symptoms in predicting pathology in patients with suspected colorectal cancer symptoms referred on a 2WW pathway: a diagnostic accuracy study

**DOI:** 10.1136/gutjnl-2020-321956

**Published:** 2020-10-21

**Authors:** Nigel D'Souza, Theo Georgiou Delisle, Michelle Chen, Sally Benton, Muti Abulafi, The NICE FIT Steering Group, Oliver Warren

**Affiliations:** 1 Colorectal Surgery, Croydon University Hospital, Croydon, UK; 2 Colorectal Surgery, Basingstoke and North Hampshire Hospital, Basingstoke, UK; 3 Surgery & Cancer, Imperial College London, London, UK; 4 Research & Development, RM Partners, London, UK; 5 Clinical Biochemistry, Royal Surrey County Hospital, Guildford, UK

**Keywords:** endoscopy, colonoscopy, colorectal cancer, stool markers, clinical decision making

## Abstract

**Objective:**

To assess whether a faecal immunochemical test (FIT) could be used to select patients with suspected colorectal cancer (CRC) symptoms for urgent investigation.

**Design:**

Multicentre, double-blinded diagnostic accuracy study in 50 National Health Service (NHS) hospitals across England between October 2017 and December 2019. Patients referred to secondary care with suspected CRC symptoms meeting NHS England criteria for urgent 2 weeks wait referral and triaged to investigation with colonoscopy were invited to perform a quantitative FIT. The sensitivity of FIT for CRC, and effect of relevant variables on its diagnostic accuracy was assessed.

**Results:**

9822 patients were included in the final analysis. The prevalence of CRC at colonoscopy was 3.3%. The FIT positivity decreased from 37.2% to 19.0% and 7.6%, respectively, at cut-offs of 2, 10 and 150 µg haemoglobin/g faeces (µg/g). The positive predictive values of FIT for CRC at these cut-offs were 8.7% (95% CI, 7.8% to 9.7%), 16.1% (95% CI 14.4% to 17.8%) and 31.1% (95% CI 27.8% to 34.6%), respectively, and the negative predictive values were 99.8% (95% CI 99.7% to 99.9%), 99.6% (95% CI 99.5% to 99.7%) and 98.9% (95% CI 98.7% to 99.1%), respectively. The sensitivity of FIT for CRC decreased at the same cut-offs from 97.0% (95% CI 94.5% to 98.5%) to 90.9% (95% CI 87.2% to 93.8%) and 70.8% (95% CI 65.6% to 75.7%), respectively, while the specificity increased from 64.9% (95% CI 63.9% to 65.8%) to 83.5% (95% CI 82.8% to 84.3%) and 94.6% (95% CI 94.1% to 95.0%), respectively. The area under the receiver operating characteristic curve was 0.93 (95% CI 0.92 to 0.95).

**Conclusion:**

FIT sensitivity is maximised to 97.0% at the lowest cut-off (2 µg/g); a negative FIT result at this cut-off can effectively rule out CRC and a positive FIT result is better than symptoms to select patients for urgent investigations.

**Trial registration number:**

ISRCTN49676259.

**Significance of this study**

**What is already known on this subject?**

- Faecal immunochemical tests (FIT) are already recommended by the National Institute for Heath and Care Excellence to guide referral of patients with low-risk bowel symptoms but has not been recommended for all symptomatic patients due to concerns over the quality and power of previous studies.

**What are the new findings?**

- FIT sensitivity for colorectal cancer (CRC) is maximised to 97.0% at the limit of detection of 2 µg haemoglobin (Hb)/g faeces (µg/g).
- A faecal Hb concentration (*f*-Hb) result less than the limit of detection in symptomatic patients indicates that their chances of not having CRC is 99.8%.
- There was no significant variation in the ability of FIT to detect CRC by patient or tumour characteristics, including age, sex, ethnicity, deprivation or iron-deficiency anaemia.

**How might it impact on clinical practice in the foreseeable future?**

- FIT could be used to rule out CRC in primary care for symptomatic patients meeting 2 weeks wait criteria, with sensitivity equivalent to colonoscopy at a cut-off of 2 µg/g.
- FIT can be used to prioritise patients for investigation, as CRC and other serious bowel disease is more likely at higher *f*-Hb concentrations.
- The diagnostic accuracy of FIT for CRC is superior to symptoms.

## Introduction

Bowel symptoms are the imprecise basis of referral for urgent investigation in England to rule out cancer.^1 2^ Symptoms are non-specific for colorectal cancer (CRC); 96 of 100 patients referred urgently on a 2-week (2WW) wait pathway under National Institute for Health and Care Excellence (NICE) NG12 guidelines will not have CRC.^1^ Urgent referrals have increased by 90% over the last 5 years^3^; 45% of UK endoscopy units are failing to meet CRC waiting targets.^4^

The faecal immunochemical test (FIT) was recommended by NICE (DG30)^2^ in 2017 to guide the referral of patients with low-risk symptoms of CRC, and is currently used in the National Health Service (NHS) of England. FIT detects the globin component of haemoglobin (Hb) by immunoassay and can reliably measure the faecal Hb concentration (*f-*Hb) to the nearest microgram of Hb per gram of faeces (µg/g).^5^ Since 2010, over 25 diagnostic accuracy studies have reported data on the use of FIT in symptomatic patients utilising a range of cut-offs.^6–8^ In 2014, a study of 787 symptomatic patients from Spain suggested that FIT is more accurate for the detection of CRC than NICE 2005 criteria (CG27) although NICE have since expanded its referral criteria to include lower risk symptoms (NG12).^9 10^ More recently, two meta-analyses reported the sensitivity of FIT for CRC in symptomatic patients at a cut-off of 10 µg/g was 92.1% (95% CI 86.9% to 95.3%)^6^ and 94.1% (95% CI 90.0% to 96.6%).^7^ However, meta-analyses cannot account for variation in *f*-Hb concentrations by patient-level variables such as age,^11–13^ sex,^11–13^ deprivation^13 14^ and between homogeneous ethnic population,^15^ which may lead to higher rates of undetected cancers within certain groups of patients. Consequently, a health technology assessment recommended that diagnostic cohort studies were performed to investigate variation in FIT accuracy in relevant subgroups.^6^ Similarly, a systematic review concluded a clear need for research on FIT as a triage test in the symptomatic primary care population.^16^

The NICE guidelines and FIT (NICE FIT) study was designed to investigate whether FIT could be used to rule out CRC in symptomatic patients in primary care meeting NICE 2WW criteria, and guide referral for further investigation.

## Methods

### Study design

The study met Standards for Reporting of Diagnostic Accuracy Studies (STARD) guidelines.^17^ Ethics and study approval were granted from the UK Health Research Authority (IRAS 218404). Patients were recruited at 50 NHS hospitals across England; sites were opened sequentially during the study.

The primary outcome measure was to identify a suitable *f*-Hb cut-off that would maximise sensitivity for CRC. The secondary outcome measures were to establish the diagnostic accuracy of FIT for CRC and other serious bowel disease (SBD) at different *f*-Hb cut-offs, and investigate the impact of other variables, such as age, sex, ethnicity and deprivation.

### Patient and public involvement

Patient and public representatives were consulted through a process of in-depth interviews during the development of the study protocol. All relevant feedback was considered and incorporated into patient information sheets. Study progress and feedback was provided regularly to the Royal Marsden Partners (RM Partners) Patient Advisory Group by the senior research manager. The chief investigator regularly reported to the RM Partners Clinical Oversight Group which includedpatient and public involvement representatives throughout all phases of the study. The results will be disseminated to trial participants directly via email and the website (https://www.nicefitstudy.com/), to other healthcare professionals at scientific conferences and through press releases.

### Patient selection

All patients referred from primary care with symptoms of suspected CRC meeting NICE referral criteria under the 2WW pathway and who were triaged by secondary care clinicians to investigation by colonoscopy were eligible for inclusion. Secondary care sites were opened continuously throughout the process. The total number of eligible patients at each site was not captured but was dependent on the volume of referrals received by each site, and the length of time the study was open to recruitment. Data on symptoms were extracted from NICE NG12 2WW and DG30 referral criteria completed on the referral form by primary care clinicians.^1 2^ Patients referred urgently on a 2WW pathway without meeting NICE criteria due to clinical concerns were classified as ‘others’ and included in the analysis. Since patients are often referred with multiple symptoms or signs, a hierarchy was created to match one criterion to each patient, based on clinical estimation of positive predictive values (PPV). NG12 criteria were ranked in importance as follows: abdominal mass, iron-deficiency anaemia (IDA) (patients over 60 years), rectal bleeding, change in bowel habit (over 60) and abdominal pain and weight loss. DG30 criteria, were ranked in importance as follows: IDA (under 60), non-IDA, abdominal pain or weight loss, change in bowel habit (under 60).

Patients were identified by the central study team or local cancer research network (CRN) team once they had been booked for colonoscopy and contacted by post or telephone and invited to participate in the study. Patients were sent an FIT specimen collection device and asked to collect one sample of faeces prior to commencing bowel preparation for their colonoscopy. A first-class return envelope was enclosed for patients to post their sample directly to the study laboratory. Patients initially provided written consent, and after approval from the National Confidentiality Advisory Group, gave implied consent by returning an FIT sample.

Patients were not included if they did not return an FIT sample, did not have a complete colonoscopy unless due to CRC, were retriaged to another investigation (eg, flexible sigmoidoscopy or CT), or withdrew consent. Patients due to undergo colonoscopy within 3 days of identification were not invited to participate in the study, as there would not have been sufficient time to return a sample. In the original NG12 guidance,^1^ NICE recommended that patients with low risk bowel symptoms were tested with a guiac-based faecal occult blood test (gFOBT) prior to 2WW referral. In many regions, these patients were referred on 2WW pathways without gFOBT due to concerns over its poor sensitivity for CRC,^18^ and therefore, were eligible for inclusion. During this study, NICE recommended that low risk patients, as defined in DG30,^2^ were triaged in primary care with FIT prior to 2WW referral. This guidance was not fully implemented during this study, but those low-risk patients who were tested with FIT in primary care prior to referral were not included.

### Index test and reference standard

FIT analysis was performed at one centralised laboratory where staff were blinded to patient clinical information. One HM-JACKarc analytical system (Hitachi Chemical Diagnostics Systems, Tokyo, Japan, supplied by Alpha Labs, Eastleigh, Hants, UK) was used to analyse all samples. The analytical working range is 7–400 µg/g. The limit of detection (LoD) of the assay is 2 µg/g and the limit of quantitation is 7 µg/g. NICE recommended an *f*-Hb cut-off of 10 µg/g in the DG30 guidelines.^2^ In accordance with previous publications on FIT, we chose the LoD and the *f*-Hb cut-off recommended in NICE DG30 as cut-offs to investigate sensitivity. To investigate the specificity and PPV at higher *f*-Hb, we also chose a higher cut-off of 150 µg/g that had previously been reported to predict high rates of significant pathology.^19^ FIT specimen collection and handling, quality management and result handling was conducted and reported according to recent guidelines for studies on FIT^20^ (see online supplemental appendix), using recommended analytical performance specifications.^5^ FIT samples that were unsuitable for analysis (collection device over or underfilled, or unavailable for analysis for more than 14 days) or performed after the colonoscopy were not included in the study.

10.1136/gutjnl-2020-321956.supp1 Supplementary data

Colonoscopy was chosen as the reference standard since it is acknowledged to be the gold-standard investigation for colorectal disease. Colonoscopists were blinded to the FIT results. Colonoscopy results were entered onto a secured online database designed specifically for the study by an external clinical research organisation (Hammersmith Medicines Research) and based on the national endoscopy logbook. Patients with incomplete colonoscopies (except when due to the presence of CRC) were excluded.

Clinical data extraction was performed initially by the local CRN team. A rigorous system of quality assurance was implemented. All colonoscopy and pathology results, as well as clinical and pathological tumour staging. were checked by the central study team, and then again by a team of senior colorectal clinicians blinded to the FIT laboratory results.

### Sample size

To determine the sample size, calculations were based on a significance level of 5%, power of 80% and prevalence of CRC within the NICE 2WW symptomatic population estimated at 3.5% based on data from the RM Partners Network. To demonstrate a lowest acceptable sensitivity of FIT for CRC of 98% with CI width of 2%, a total sample size of 5379 patients was required. Given that previous studies had reported a 50% non-completion rate, it was determined that at least 10 000 patients would need to be invited to participate in the study. The study was funded to over-recruit beyond this sample size to address the secondary endpoints and investigate the impact of other factors on FIT diagnostic accuracy. Accurate power calculations were not possible for the secondary endpoints, due to the lack of data on these covariates on the diagnostic accuracy of FIT for CRC in the symptomatic populations.

### Data analysis

Patients with multiple findings at colonoscopy were recategorised with one diagnosis in a hierarchy; CRC ranked highest followed by high-risk adenoma (HRA) and then inflammatory bowel disease (IBD). These were grouped together as SBD. This was followed by low-risk adenoma (LRA) which was ranked above other non-malignant diagnoses, including diverticular disease, microscopic colitis, benign perianal disease (haemorrhoids, anal fissures, anal fistulas, solitary rectal ulcers), angiodysplasia, or rare findings such as melanosis coli, parasites or lipomas. HRA was defined by the NICE FIT Steering group as any polyp with high-grade dysplasia or polyps over 10 mm in size with low grade dysplasia, and serrated lesions in the right colon. Other polyps less than 10 mm were classified as LRA.

The indices of multiple deprivation were derived from postcodes (1=most deprived and 10=least deprived).^21^ Patients were classified as anaemic according to WHO criteria^22^; blood Hb concentration less than 120 g/L for women or 130 g/L for men, based on the most recent measurement within 3 months before referral. IDA was defined using British Society of Gastroenterology guidelines as present when serum ferritin concentration was less than 15 µg/L.^23^

Data were assessed for normality by the Shapiro test and Q-Q plot analysis. Mann-Whitney and Kruskal Wallis tests were used for non-normally distributed data. Analysis of variancewas used across multiple groups, with separate models for each factor; age was pooled for analysis. Categorical data were compared with χ^2^ tests. Sensitivity, specificity, PPV and negative predictive value (NPV) were reported for each *f-*Hb cut-off, with 95% CIs. Receiver operating characteristic (ROC) curves were plotted for *f*-Hb. These were done using an initial threshold of 0.1 to calculate sensitivity and specificity, and then recalculated with increments of 0.1 to plot the ROC curve. In every statistical analysis, p<0.05 was considered significant. All analyses were performed using SAS V.9.4 (SAS Institute).

## Results

**Figure 1:**
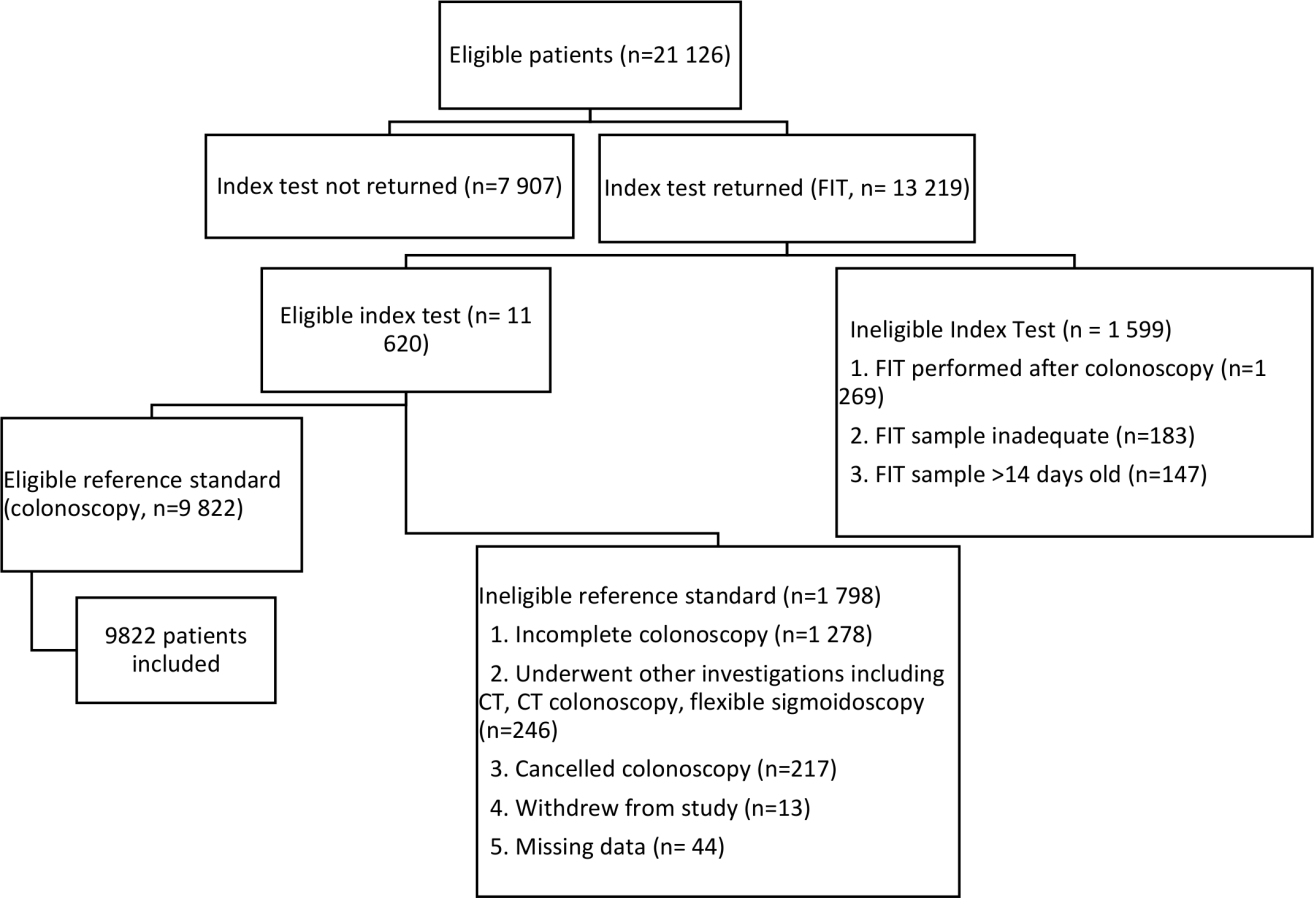
NICE FIT study flow diagram (adapted from STARD). FIT, faecal immunochemical test; NICE, National Institute for Heath and Care Excellence; STARD, Standards for Reporting of Diagnostic Accuracy Studies.

Between October 2017 and December 2019, 21 126 patients were sent recruitment packs, 13 219 (62.6%) returned FIT devices. Complete FIT and colonoscopy outcomes were available for 9 822 patients, who were included in the study results. A study flow diagram is shown in figure 1: NICE FIT study flow diagram (adapted from STARD). Data were not uploaded by the local sites for 44 patients were excluded.

Patient demographics are summarised in table 1. The median patient age was 65.0 years (IQR 56.0–73.0). Women returned 54.9% of kits. The most common ethnic groups were white (75.9%), other (11.2%) and Asian (6.3%). The median deprivation index score was 6.0 (IQR 4.0–9.0). Patients were referred most commonly with high-risk symptoms meeting NG12 criteria (73.2%), followed by low-risk symptoms meeting DG30 criteria (21.4%) or other symptoms warranting urgent referral (6.4%).

**Table 1:** Patient demographics

	N	%
Total	9822	100
Sex		
Women	5394	54.9
Men	4428	45.1
Age (years)		
Mean	64.0	
SD	11.9	
Minimum	17	
Median	65	
Maximum	97	
Age group (years)		
<40	361	3.7
41–50	940	9.6
51–60	2226	22.7
61–70	3033	30.9
>70	3262	33.2
Ethnicity		
White	7453	75.9
Asian	614	6.3
Black	365	3.7
Mixed	58	0.6
Chinese	42	0.4
Other*	1103	11.2
Index of deprivation		
Mean	6.22	
SD	2.62	
Median	6	
Symptom risk category		
High-medium (NG12)	7194	73.2
Low (DG30)	1994	20.3
Other**†**	634	6.5

*Other ethnicity: any other ethnic group, not-specified.

†Other symptoms: patients referred urgently with symptoms of suspected CRC not meeting existing NG12 or DG30 criteria.

CRC, colorectal cancer.

Tests that were older than 14 days or sampled inadequately (n=330) could not be analysed. FIT analysis was performed within 7 days of sample collection in 94.8% of specimens, and within a day of receipt by the laboratory in 94.6% of specimens.

Findings at colonoscopy are reported in table 2. Overall, the most prevalent finding at colonoscopy was that no disease was detected (31.3%). SBD (CRC, HRA or IBD) was detected in 11.9% of patients during colonoscopy. CRC was detected in 3.3% of patients.

**Table 2:** Frequency of pathology findings at colonoscopy in symptomatic patients referred via 2WW pathways

Diagnosis	N	%
Normal	3079	31.3
Low risk adenoma	2321	23.6
Diverticular disease	2294	23.4
Perianal disease*	723	7.4
Inflammatory bowel disease	427	4.3
High-risk adenoma	421	4.3
Colorectal cancer	329	3.3
Microscopic colitis	152	1.5
Other†	53	0.5
Angiodysplasia	23	0.2

*Perianal disease: anal fissure, anal fistula, haemorrhoids or solitary rectal ulcer.

†Other: findings included melanosis Coli, parasites, lipoma.

2WW, 2 weeks wait.

**Figure 2:**
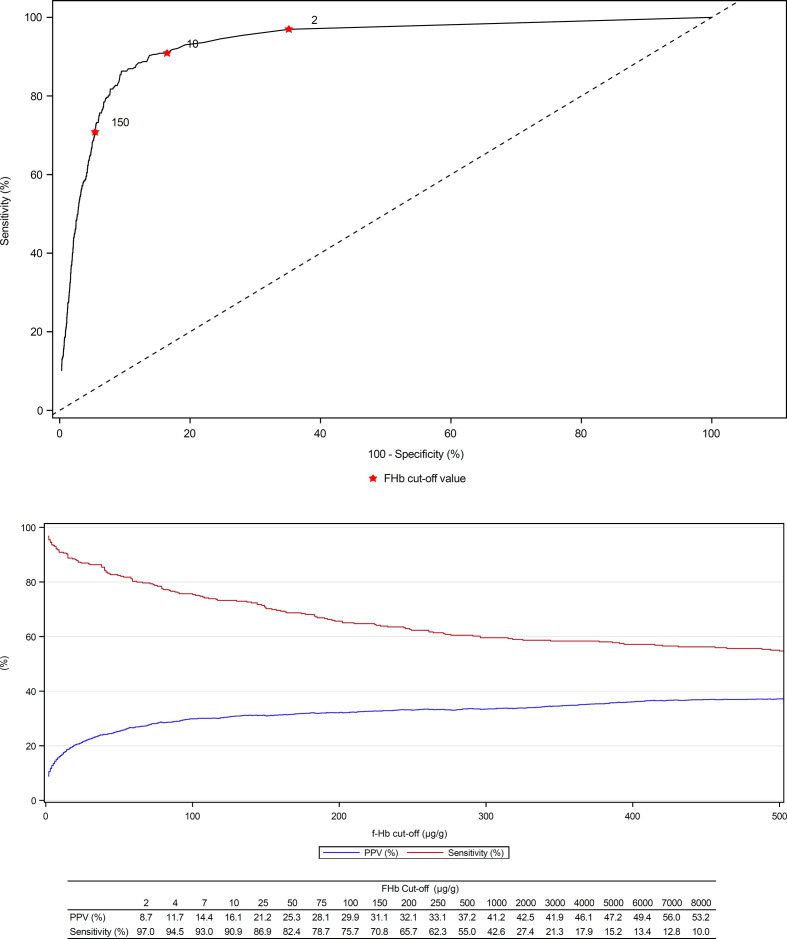
ROC curve (top) and PPV/sensitivity (bottom) of FIT for CRC. CRC, colorectal cancer; FIT, faecal immunochemical test; PPV, positive predictive value; ROC, receiver operating characteristic.

The diagnostic accuracy of FIT for CRC at *f-*Hb cut-offs of 2 µg/g, 10 µg/g and 150 µg/g are summarised in table 3. The proportion of patients that had positive FIT results at *f*-Hb cut-offs of 2, 10 and 150 µg/g, respectively, decreased significantly (p<0.0001) from 37.2% to 19.0% and 7.6%, respectively. At the same cut-offs, the PPV for CRC increased from 8.7% (95% CI, 7.8% to 9.7%) to 16.1% (95% CI 14.4% to 17.8%) and 31.1% (95% CI 27.8% to 34.6%), but the sensitivity for CRC declined from 97.0% (95% CI 94.5% to 98.5%) to 90.9% (95% CI 87.2% to 93.8%) and 70.8% (95% CI 65.6% to 75.7%), as illustrated in figure 2. The number needed to scope,^24^ that is, number of individuals required to undergo colonoscopy to detect 1 CRC was 11.5, 6.2 and 3.2 at *f*-Hb cut-offs of 2, 10 and 150 µg/g, compared with 29.9 for all patients referred on the current 2WW pathway. Some CRCs were not detected even a cut-off of 2 µg/g, but significantly more CRCs (30 vs 10, p=0.0011) were not detected at a cut-off of 10 µg/g. When f-Hb was undetectable (<2 µg/g), the PPV for CRC was 0.2% (0.1%–0.3%), and 617 patients would require colonoscopy to detect 1 CRC.

**Table 3:** Diagnostic accuracy of FIT for CRC at different cut-offs

Cut-off (µg/g)	Positivity (%)	NNS	Sensitivity (%)	Specificity (%)	PPV (%)	NPV (%)	TP	FN	FP	TN
2	37.2	11.5	97.0 (94.5 to 98.5)	64.9 (63.9 to 65.8)	8.7 (7.8 to 9.7)	99.8 (99.7 to 99.9)	319	10	3336	6157
10	19.0	6.2	90.9 (87.2 to 93.8)	83.5 (82.8 to 84.3)	16.1 (14.4 to 17.8)	99.6 (99.5 to 99.7)	299	30	1563	7930
150	7.6	3.2	70.8 (65.6 to 75.7)	94.6 (94.1 to 95.0)	31.1 (27.8 to 34.6)	98.9 (98.7 to 99.1)	233	96	516	8977
<2	62.8	616.7	3 (1.5 to 5.5)	35.1 (34.2 to 36.1)	0.2 (0.1 to 0.3)	91.3 (90.3 to 92.2)	10	319	6157	3336

95% CIs within brackets.

CRC, colorectal cancer; FIT, faecal immunochemical test; FN, false negatives; FP, false positives; NNS, number needed to scope; NPV, negative predictive value; PPV, positive predictive value; TN, true negatives; TP, true positives.

The diagnostic accuracy of FIT for SBD is summarised in table 4. The sensitivity of FIT for HRA and IBD are significantly lower than for CRC at every *f-*Hb cut-off. The PPV for SBD increases significantly at higher *f*-Hb cut-offs; 24.8% at 2 µg/g, 39.6% at 10 µg/g and 64.5% at 150 µg/g.

**Table 4:** Diagnostic accuracy of FIT for CRC and SBD at different cut-offs

Risk category	FIT positivity	Cut-off (µg/g)	Sensitivity	Specificity	PPV	NPV
≥2	37.2	CRC	97.0 (94.5 to 98.5)	64.9 (63.9 to 65.8)	8.7 (7.8 to 9.7)	99.8 (99.7 to 99.9)
		HRA	65.8 (61.0 to 70.3)	64.1 (63.1 to 65.0)	7.6 (6.7 to 8.5)	97.7 (97.3 to 98.0)
		IBD	73.1 (68.6 to 77.2)	64.4 (63.4 to 65.4)	8.5 (7.7 to 9.5)	98.1 (97.8 to 98.5)
		SBD	77.1 (74.6 to 79.5)	68.2 (67.2 to 69.2)	24.8 (23.4 to 26.3)	95.6 (95.1 to 96.1)
≥10	19.0	CRC	90.9 (87.2 to 93.8)	83.5 (82.8 to 84.3)	16.1 (14.4 to 17.8)	99.6 (99.5 to 99.7)
		HRA	45.4 (40.5 to 50.3)	82.2 (81.4 to 83.0)	10.3 (8.9 to 11.7)	97.1 (96.7 to 97.5)
		IBD	57.8 (53.0 to 62.6)	82.8 (82.0 to 83.6)	13.3 (11.8 to 14.9)	97.7 (97.4 to 98.1)
		SBD	62.6 (59.8 to 65.4)	87.0 (86.3 to 87.7)	39.6 (37.4 to 41.8)	94.5 (93.9 to 95.0)
≥150	7.6	CRC	70.8 (65.6 to 75.7)	94.6 (94.1 to 95.0)	31.1 (27.8 to 34.6)	98.9 (98.7 to 99.1)
		HRA	22.1 (18.2 to 26.4)	93.0 (92.5 to 93.5)	12.4 (10.1 to 15.0)	96.4 (96.0 to 96.8)
		IBD	36.8 (32.2 to 41.5)	93.7 (93.2 to 94.2)	21.0 (18.1 to 24.1)	97.0 (96.7 to 97.4)
		SBD	41.0 (38.2 to 43.9)	96.9 (96.5 to 97.3)	64.5 (60.9 to 67.9)	92.4 (91.8 to 92.9)

95% CIs within brackets.

CRC, colorectal cancer; FIT, faecal immunochemical test; HRA, high-risk adenoma; IBD, inflammatory bowel disease; NPV, negative predictive value; PPV, positive predictive value; SBD, serious bowel disease.

On ROC curve analysis (figure 2), the area under the curve (AUC) for CRC was 0.93 (0.92–0.95). Youden’s index, which maximises the sum of sensitivity and specificity was 38 µg/g, but FIT sensitivity was still optimised at 2 µg/g.

Patients with CRC that had *f-*Hb <10 µg/g were analysed in further detail (table 5). There were no significant differences between patients with CRC and *f-*Hb greater or less than either cut-off of 2 µg/g or 10 µg/g with regard to age, sex, deprivation or ethnicity, iron and non-IDA or tumour characteristics.

**Table 5:** Characteristics of 329 CRCs diagnosed in patients referred on a 2WW pathway overall, and classified by false negative FIT results at cut-offs of 2 or 10 µg/g

Variable	n	%	*f*-Hb cut-off		P value	*f*-Hb cut-off		P value
			≤2 µg/g	>2 µg/g		≤10 µg/g	>10 µg/g	
Sex								
Female	130	39.5	4	126	0.98	15	115	0.22
Male	199	60.5	6	193		15	184	
Age group (years)								
30–49	16	4.9	2	14	0.17	3	13	0.51
50–59	65	19.8	3	62		5	60	
60–69	87	26.4	2	85		6	81	
70–79	117	35.6	2	115		13	104	
80+	44	13.4	1	43		3	41	
Ethnicity								
Asian	15	4.6	0	15	0.87	2	13	0.87
Black	8	2.4	0	8		0	8	
Chinese	1	0.3	0	1		0	1	
Mixed	2	0.6	0	2		0	2	
Other	34	10.3	0	34		2	32	
White	266	80.9	8	258		24	242	
Missing	3	0.9	2	1		2	1	
Deprivation								
1	6	1.8	0	6	0.51	1	5	0.13
2	17	5.2	1	16		1	16	
3	26	7.9	0	25		0	26	
4	39	11.9	3	36		7	32	
5	36	10.9	2	34		5	31	
6	40	12.2	0	40		2	38	
7	41	12.5	0	41		0	41	
8	42	12.8	1	41		5	37	
9	40	12.2	2	38		5	35	
10	42	12.8	1	41		4	38	
Tumour morphology								
Polypoid	163	49.5	6	157	0.48	16	147	0.66
Ulcerated	132	40.1	3	129		11	121	
Missing	34	10.3	1	33		3	30	
Tumour subtype								
Adenocarcinoma	269	86.9	9	277	0.28	28	286	0.74
Mucinous	9	2.7	1	8		1	8	
Other	21	6.4	0	21		1	20	
Missing	13	4.0	0	13		0	13	
Luminal narrowing								
No	103	31.3	3	100	0.73	12	91	0.26
Passable	114	34.7	5	109		12	102	
Impassable	81	24.6	2	79		4	77	
Missing	81	24.6	0	31		2	20	
Site								
Caecum	30	9.1	1	29	0.19	6	24	0.16
Ascending Colon	45	13.7	1	44		5	40	
Hepatic Flexure	15	4.6	2	13		3	12	
Transverse Colon	14	4.3	1	13		1	13	
Splenic Flexure	5	1.5	1	4		1	4	
Descending Colon	8	2.4	0	8		2	6	
Sigmoid Colon	60	18.2	2	58		4	56	
Rectosigmoid	23	7.0	0	23		1	22	
Rectum	112	34.0	2	110		6	106	
Anus	5	1.5	0	5		1	4	
Missing	12	3.7	0	12		0	12	
N stage								
1	18	6.1	1	19	0.98	3	17	0.31
2	64	19.5	2	62		10	54	
3	182	55.3	6	176		14	168	
4	41	12.5	1	40		3	438	
Unknown	4	1.2	0	4		0	4	
Missing	18	5.5	0	18		0	18	
N stage								
0	158	53.2	4	154	0.71	16	142	0.44
1	83	25.2	4	79		11	72	
1c	26	7.9	0	26		1	25	
2	43	13.1	2	41		2	41	
Missing	19	5.8	0	19		0	19	
Anaemia								
Yes	128	38.9	2	126	0.65	7	121	0.13
No	173	52.6	4	165		18	155	
Missing	28	8.5	4	28		5	23	
Iron deficiency anaemia								
Yes	73	20.4	1	72	0.63	4	69	0.22
No	173	51.4	4	169		18	155	
Missing	83	28.3	5	78		8	75	

Measures of association assessed by χ^2^.

CRC, colorectal cancer; FIT, faecal immunochemical test; 2WW, 2 weeks wait.

## Discussion

This is the first powered, multicentre, double-blinded diagnostic accuracy study to demonstrate that FIT can be used to select patients with NICE 2WW symptoms for urgent investigation. FIT can be used to rule out CRC when *f*-Hb is undetectable or low. FIT sensitivity for CRC is significantly higher at 97% when using a lower *f-*Hb cut-off of the LoD (2 µg/g) compared with 10 µg/g, the cut-off recommended in NICE DG30. No significant difference was found in FIT sensitivity on subgroup analysis by age, sex, deprivation, ethnicity and tumour characteristics, suggesting FIT can be used in all symptomatic patients that meet 2WW referral criteria. Employing a higher cut-off for investigation will result in a smaller group of FIT positive patients with a higher PPV or prevalence for CRC, but at the expense of detecting fewer CRC; this strategy may be adopted when endoscopy capacity is restricted or paused as occurred at the height of the current COVID-19 pandemic.^25 26^ The likelihood of cancer increases with increasing *f*-Hb concentrations (above 150 µg/g), and consequently, FIT could be used to rule-in cancer or prioritise patients for investigation.

The most common finding at colonoscopy in symptomatic patients in our study was the absence of disease (31.3%) in keeping with other reports on 2WW referrals^19 27^; FIT can appropriately triage these patients off urgent pathways for investigation. Importantly, a negative FIT result can be used to reassure patients that their symptoms are unlikely to be due to CRC because of the high NPV; 99.8% and 99.6% at 2 µg/g and 10 µg/g, respectively. Patients with symptoms meeting NICE criteria and a negative FIT result at these cut-offs have less than 0.5% chance of CRC; a very low risk, but not no risk. In patients with undetectable *f*-Hb, 617 patients would need to undergo colonoscopy to detect 1 CRC; hence clinical acumen and safety-netting remains essential to identify patients with CRC and false negative FIT.^28^ The ROC AUC of 0.93 (0.92–0.95) confirms that the diagnostic accuracy of FIT is excellent, and on its own is at least as good as risk scores such as FAST (AUC 0.91) or COLONPREDICT (AUC 0.92) that combine FIT with other patient characteristics such as demographics, serum Hb and symptoms.^29 30^

Colonoscopy currently remains the gold-standard investigation to diagnose or exclude CRC but can fail to detect CRC. The sensitivity of colonoscopy for CRC in a meta-analysis of 9223 patients in 25 studies was 94.7% (95% CI 90.4% to 92.7%), although the largest trials reported data from asymptomatic participants in screening programmes.^31^ A recent study from the UK reported that the postcolonoscopy CRC rate at 3 years was 3.6%–7.4%, implying that these CRC were potentially not detected at index colonoscopy.^32^ In this context, FIT sensitivity of 97.0% for CRC at a cut-off of the LoD appears to be equivalent to colonoscopy for the detection of CRC. Other SBDs such as HRA and IBD are associated with a raised *f*-Hb; the PPV of 64.5% for SBD at 150 ug/g is clinically significant. However, the poor sensitivity of FIT for HRA at 65.8% and IBD at 73,1% at a cut-off of 2% and 45.4% and 57.8% at a cut-off of 10 suggest that FIT does not reliably identify these conditions.

FIT has already been recommended by NICE DG30 to triage patients with low-risk symptoms^2^ for investigation, but at the time was not been recommended for high-risk symptoms, due to a lack of robust evidence within the UK setting and because *f-*Hb are known to vary by age,^11–13^ sex,^11–13^ deprivation,^13 14^ cancer stage,^33^ IDA^19 27^ and between homogeneous ethnic populations.^15^ We investigated these known covariates and found that there was no significant difference in FIT sensitivity for CRC across all groups including cancer stage and IDA at cut-offs of 2 and 10 µg/g. Previous studies have reported some differences in median *f-*Hb across these variables^11–15^ but this was not clinically relevant for detection of CRC at the different cut-offs investigated. We have not found significant difference in FIT sensitivity in patients referred with IDA, as was reported in other studies.^19 27^ However, missing data during referral from primary care or even prior to colonoscopy was common, particularly ferritin concentration (30%), but even Hb. Furthermore, data on luminal narrowing was not reported in 24.6% of colonoscopy reports, and while ethnic representation reflected the UK population, the numbers of some minority groups even within a study of this size remained small, and the potential for type II error exists.

Although not yet recommended by NICE, FIT is already being used by some services for high-risk symptoms. The largest two reports on the diagnostic accuracy of FIT in high risk symptoms were from service evaluations within Nottingham^19^ in England and NHS Tayside in Scotland.^27^ Neither study investigated the impact of age, sex, deprivation or ethnicity on FIT diagnostic accuracy. Chapman *et al* 19 investigated 1106 patients in Nottingham with NICE NG12 2WW symptoms (excluding rectal bleeding) with FIT. Similar results to our study were reported, with sensitivity for CRC of 97.5%, 87.5% and 60% at cut-offs of 4 µg/g (the LoD of the FIT system used), 10 µg/g and 150 µg/g, respectively; the PPV for CRC at the same cut-offs were 12.5%, 14.6% and 35.8%, respectively. No disease was found at colonoscopy in 58.8% of patients in Nottingham. In NHS Tayside, Mowat *et al* 27 reported the results on 1447 symptomatic patients investigated with FIT prior to colonoscopy. At a cut-off of 10 µg/g, the sensitivity of FIT was 90.5% and PPV was 11.0%: no disease was found in 27.8% of patients. FIT sensitivity for CRC in our study at 10 µg/g was similar to the results of previous meta-analyses of 4091 symptomatic patients 92.1% (95% CI 86.9% to 95.3%)^6^ and 6698 patients with specifically high-risk symptoms 91.7% (95% CI 83.3% to 96.1%).^7^ However, given this prospective, multicentre research study is the largest to date, and designed to meet the highest methodological quality using STARD guidelines, our results on FIT accuracy unequivocally supports the use of FIT as a basis to triage patients with 2WW symptoms for referral and investigation.

Our system of quality assurance is the first described in the symptomatic FIT literature; over 30% of errors in colonoscopy data coding were detected by clinicians, including misclassification of CRC. The missed CRC rate is unknown, since the volume and key performance indicators of individual endoscopists are unknown, although the majority of endoscopy units in this study were accredited by the Joint Advisory Group on Gastrointestinal Endoscopy. As 11% of colonoscopies were incomplete and excluded from analysis, it is possible that the true prevalence of pathology present in a 2WW population was not captured by this study but at 3.3%, CRC prevalence in this study is equivalent to CRC prevalence in the 2WW population recorded nationally.^3 34^

We found no obvious pattern or cause for false negative FIT results in patients with CRC, which may require further research into patient-level (genetic or medication) variables. Sampling studies in symptomatic patients (eg, multiple samples from the one bowel motion or consecutive motions) may provide possible strategies to improve sensitivity. Sequential use of further biomarkers (including volatile organic compounds in the urine, faeces or breath) following FIT might reduce the number of false positive and false negative results.^35 36^ Our diagnostic accuracy results may not be replicated in other laboratories or FIT analysers, which may not be able to detect *f-*Hb down to 2 µg/g; an international group is working on FIT method standardisation.^37^

Finally, the optimal FIT pathway remains unclear. When FIT was used in primary care in Scotland, referrals reduced by 15.1%.^27^ When FIT accompanied referral in Nottingham, 2WW referrals and 2WW CTC usage increased while there was no long-term reduction in 2WW colonoscopy usage; possibly due to referral of a wider, lower risk group of patients.^38^ We would recommend incorporating FIT into referral pathway of symptomatic patients in primary care with appropriate safety netting, to reduce unnecessary referrals for investigations and help secondary care prioritise patients with higher risk of CRC. NICE have already recommended in their DG30 guidance use of FIT in primary care as a triaging tool for low risk symptoms before referral to secondary care; this strategy should be expanded to include all symptomatic patients. The *f*-Hb cut-off for onwards referral should be set at the LoD (2 µg/g) to provide sensitivity equivalent to colonoscopy, the current gold standard for investigation and yet reduce referrals by 60%. While not the primary intention of the 2WW pathway, more cases of HRA (20.4%) and IBD (15.3%) will also be detected at the LOD than 10 µg/g. Alternatively, the *f*-Hb cut-offs could be set higher to reduce referrals further to match existing colonoscopy resource and maximise the PPV for CRC.

## Conclusion

FIT is superior to 2WW symptoms in predicting pathology in patients with suspected CRC. At a cut-off of the LoD of the FIT analytical system used, FIT detects CRC with equivalent diagnostic accuracy to colonoscopy. A higher *f*-Hb cut-off may be set to match capacity in resource-limited settings; this will reduce the number of positive results, onwards referral for investigation and demand for colonoscopy but at the expense of detecting fewer cancers. High *f*-Hb levels are associated with a high PPV for CRC and SBD and can be used to prioritise investigations.

## Data Availability

No data are available.
