# Supplementary material for: Faecal immunochemical test is superior to symptoms in predicting pathology in patients with suspected colorectal cancer symptoms referred on a 2WW pathway: a diagnostic accuracy study
Source: Gut. 2020 Oct 21;70(6):1130–8. doi: 10.1136/gutjnl-2020-321956 (PMC8108285; doi:10.1136/gutjnl-2020-321956)
Supplement: Supplementary data [file gutjnl-2020-321956supp001.pdf]

Appendix 1

Specimen collection and handling

Potential participants were given a single specimen collection device (EXTEL HEMO-AUTO MC Collection Picker, Hitachi Chemical Diagnostics Systems Co., Ltd, Tokyo, Japan) from a single manufacturing lot (supplied by Alpha Labs Ltd, Eastleigh, Hants, UK) in the study recruitment pack by post.

The device collects 2 mg faeces with an indented probe integral to the cap of the device into 2.0 ml buffer. Participants were instructed to scrape the probe into the faeces passed into the toilet onto paper that was not submerged in water, as per manufacturer's instructions.

Participants completed the collection and wrote the date of sample collection on the device label. They were asked to perform the test prior to commencing bowel preparation for colonoscopy and return their kit in a pre-paid 1st class envelope addressed to the study laboratory at the NHS Bowel Cancer Screening Southern England Hub at the Royal Surrey County Hospital, Guildford. Once received at the laboratory samples were stored at 4°C (in a fridge) until analysis.

Analysis

Specimens were allowed to come to room temperature and mixed by inversion prior to analysis. One HM-JACKarc analytical system ((Hitachi Chemical Diagnostics Systems Co., Ltd, Tokyo, Japan, supplied by Alpha Labs Ltd, Eastleigh, Hants, UK)) was used to analyse all samples. Each sample was analysed once. The analytical working range is 7-400 µg/g. Samples with *f*-Hb above the upper limit of the measurement range were diluted 1 in 20 with buffer from the sampling devices. Any results >8 000 µg/g were reported as >8 000 µg/g. The LoD of the method is 2 µg/g. This was defined according to recently published recommendations. Results with this cut-off were compared to the DG30 recommended cut-off of 10 µg/g and a published recommendation of 150 µg/g. Calibrators were manufactured by Hitachi Chemical Diagnostics Systems Co., Ltd and supplied by Alpha Labs Ltd. Four calibrator lots were used, each with a pre-defined 7-point calibration, which was calibrated locally with a 2-point calibration.

Quality management

All analyses were overseen by a Health & Care Professions Council state registered biomedical scientist trained on the FIT system by Alpha Labs Ltd. The analyser was calibrated weekly with the material provided by Hitachi Chemical Diagnostics Systems Co., Ltd. Two levels of quality control material (QC) (Hitachi Chemical Diagnostics Systems Co., Ltd)) were used; target ranges were

assigned by analysing each QC material 20 times over 10 days, calculating the mean ± 2 standard deviations (SD): if any results fell outside of ± 3 SD, the assigned mean for the QC material was recalculated and the criteria for acceptance or rejection of runs modified. Both concentrations of QC were analysed before and after each run of samples from patients. Analyses involving samples from patients were accepted if the QC results fell within the assigned range, but rejected if two consecutive results fell between ± 2 and 3 SD.

Result handling

The *f*-Hb results were recorded electronically in µg/g by the analysers, within the measurement range specified above. The date of sample collection was added to these results by the laboratory staff, and then sent to the research team.
